# The gut signals to AGRP-expressing cells of the pituitary to control glucose homeostasis

**DOI:** 10.1172/JCI164185

**Published:** 2023-04-03

**Authors:** Shun-Mei Liu, Bruno Ifebi, Fred Johnson, Alison Xu, Jacquelin Ho, Yunlei Yang, Gary Schwartz, Young Hwan Jo, Streamson Chua

**Affiliations:** 1 Department of Medicine,; 2 Department of Neuroscience, and; 3 Department of Molecular Pharmacology, Albert Einstein College of Medicine, New York, New York, USA.

**Keywords:** Endocrinology, Metabolism, Glucose metabolism, Neuroendocrine regulation

## Abstract

Glucose homeostasis can be improved after bariatric surgery, which alters bile flow and stimulates gut hormone secretion, particularly FGF15/19. FGFR1 expression in AGRP-expressing cells is required for bile acids’ ability to improve glucose control. We show that the mouse *Agrp* gene has 3 promoter/enhancer regions that direct transcription of each of their own AGRP transcripts. One of these *Agrp* promoters/enhancers, *Agrp-B,* is regulated by bile acids. We generated an *Agrp-B* knockin FLP/knockout allele. AGRP-B–expressing cells are found in endocrine cells of the pars tuberalis and coexpress diacylglycerol lipase B — an endocannabinoid biosynthetic enzyme — distinct from pars tuberalis thyrotropes. AGRP-B expression is also found in the folliculostellate cells of the pituitary’s anterior lobe. Mice without AGRP-B were protected from glucose intolerance induced by high-fat feeding but not from excess weight gain. Chemogenetic inhibition of AGRP-B cells improved glucose tolerance by enhancing glucose-stimulated insulin secretion. Inhibition of the AGRP-B cells also caused weight loss. The improved glucose tolerance and reduced body weight persisted up to 6 weeks after cessation of the DREADD-mediated inhibition, suggesting the presence of a biological switch for glucose homeostasis that is regulated by long-term stability of food availability.

## Graphical abstract

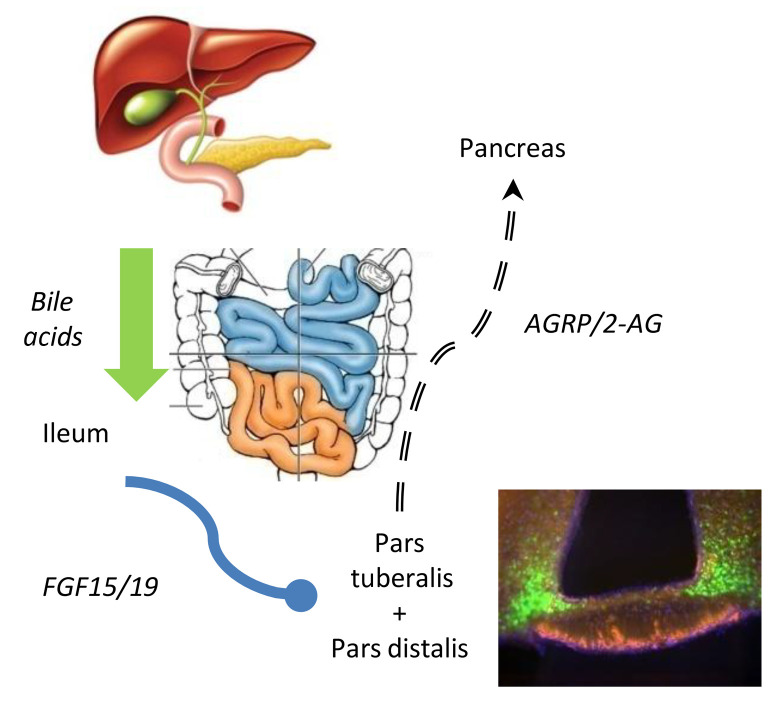

## Introduction

The hypothalamic AGRP/NPY neuron is well known to be orexigenic (1, 2), opposing the action of its counterpart, the hypothalamic anorexigenic POMC neuron (3, 4). However, constitutive absence of AGRP or NPY has little or no impact on feeding or body weight gain on normal chow (5). Only mice with adult ablation of AGRP/NPY neurons show hypophagia (6, 7), indicating that developmental compensation occurs to normalize food intake in the absence of either orexigenic peptide. We have shown that the constitutive absence of AGRP does have effects on glucose homeostasis and the reproductive system (8, 9). *Agrp*-KO mice have improved glucose tolerance if they are obese genetically as a result of leptin signaling deficiency. *Agrp*-KO females also have earlier puberty onset, while *ob/ob* *Agrp*-KO females are fertile. In addition, mammary gland development is restored in *ob/ob Agrp*-KO females. These were surprising results since severe hypoleptinemia is associated with infertility and failure to undergo puberty (10–12).

We have previously described a gut-to-brain axis (13, 14) that comprises meal-associated bile acid secretion and FGF15/19 signaling which regulates the expression and activity of AGRP-expressing cells. This signaling pathway could constitute a physiological role for AGRP in glucose homeostasis. This pathway may also be relevant to bariatric surgery (15), wherein procedures result in altered bile flow, increased FGF19 concentrations, and improved glucose control, sometimes with remission of preexisting type 2 diabetes mellitus (16, 17). In this report, while searching for potential sites of regulation within the *Agrp* upstream regulatory region, we found that public databases had identified multiple AGRP mRNA variants. The mouse *Agrp* gene has 3 widely separated transcription start sites that result in 3 different transcripts (AGRP-A, AGRP-B, and AGRP-C), which differ in their 5′-untranslated regions (5′-UTRs), implying the existence of 3 different promoters/enhancers and varying modes of regulation for each of the transcript variants. In addition, the cell type specificities of these promoters/enhancers are likely to be different. We show that the AGRP-B mRNA variant is suppressed by bile acids but remains unaffected by fasting. In contrast, the AGRP-A variant, the most proximal variant, is regulated by fasting but remains unaffected by bile acid treatment. Previously, we had shown that bile acids and FGF19 both affected glucose metabolism by improving insulin sensitivity and glucose-stimulated insulin secretion (GSIS) in lean mice on normal chow, DIO (diet-induced obesity) mice, and obese leptin-deficient (*ob/ob*) mice (13, 14). This improved glucose metabolism is dependent on FGFR1 signaling in AGRP-expressing cells as well as downstream MC4R (13), since Cre-mediated deletion of *Fgfr1* in AGRP-expressing cells abolished the glucose homeostatic response to taurocholate.

In order to determine whether AGRP-B–expressing cells are involved in glucose homeostasis, we generated a knockin/knockout allele of the mouse *Agrp* gene that expresses FLP recombinase from the *Agrp-B* promoter, *Agrp-B-FLP*. Using the *Agrp-B-FLP* allele in combination with an FLP-dependent tdTomato reporter as a lineage tracker, we found that the *Agrp-B* promoter is expressed in endocrine, non-neuronal cells of the pars tuberalis, colocalizing with PITX1 (18) and CGA, markers for adenohypophyseal cells derived from Rathke’s pouch. The *Agrp-B* promoter expression does not colocalize with cholecystokinin, which is coexpressed with TSHB in thyrotropes (19) of the pars tuberalis. The AGRP-B–expressing cells do coexpress diacylglycerol lipase B (DAGLB), a terminal enzyme in the biosynthesis of the endocannabinoid 2-arachidonylglycerol (2-AG). This expression pattern marks the AGRP-B cells as a distinct population of the pars tuberalis. We also found expression of AGRP-B FLP in folliculostellate cells of the anterior lobe of the pituitary gland with the AGRP-B cells showing extensive colabeling with S100B and SOX2.

We assessed the physiological role of the AGRP-B–expressing cells through an intersectional chemogenetic approach as well as the conventional knockout strategy. Using a combination of *Agrp-B-FLP* and *Agrp-IRES-Cre* to drive expression of an inhibitory DREADD (designer receptor exclusively activated by designer drugs), hM4Di, we examined the effect of repressing AGRP-B–expressing cells. A 5-day course of DREADD ligand–mediated suppression caused significant improvements in glucose tolerance and GSIS that persisted for 6 weeks after termination of the inhibitor treatment. Furthermore, mice that lacked AGRP-B expression were protected from glucose intolerance associated with high-fat diet but gained equivalent amounts of weight compared with wild-type mice.

Our experimental data point to a physiological role for AGRP-B–expressing cells in the regulation of glucose tolerance and insulin secretion. These cells are regulated by nutritional signals from the gut via a bile acid–FGF15/19 pathway, which in turn is regulated by the timing and contents of meals. The time scale by which AGRP-B regulates metabolism occurs over days and weeks, whereas the previously described actions of AGRP on feeding, acting via hypothalamic AGRP/NPY neurons that express AGRP-A, occur within minutes of activation. We discuss the potential mechanisms by which AGRP-B–expressing neuroendocrine cells might regulate glucose homeostasis.

## Results

### The mouse Agrp gene has 3 transcription start sites as revealed by 3 AGRP mRNA variants.

**Figure 1:**
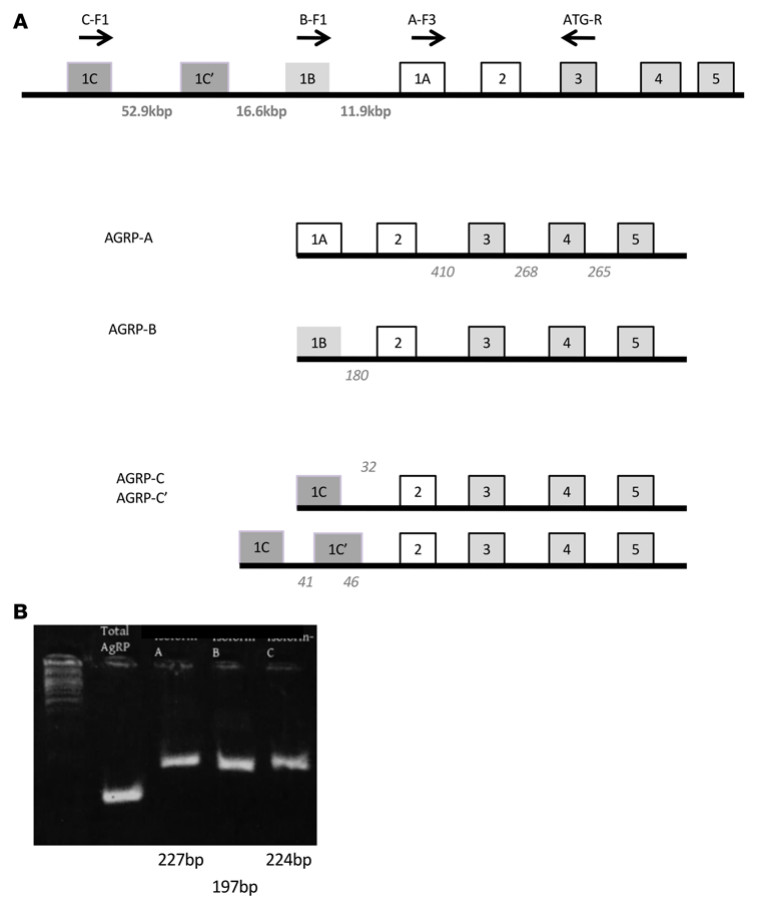
Structure of the *Agrp* gene and mRNA variants. (**A**) The exon-intron structure is shown schematically for the *Agrp* gene and its mRNA variants. Coding exons are shaded in gray. Each of the first exons (1A, 1B, and 1C) is presented in a different shade. The size of each intron is indicated below the intron. Each mRNA variant is presented with its included exons. Note that transcript C has an alternatively spliced exon (1C′). The number of spliced transcripts found from RNA-Seq studies, as reported in the NCBI Genome Data Viewer, is presented in italic text at the intron positions. Unfortunately, there are no data regarding the number of reads corresponding to intron-spanning reads that include exon A. (**B**) Results of mRNA variant–specific amplification from hypothalamic block RNA isolated from an adult male mouse. Total AGRP represents amplification for all AGRP mRNA variants, and each subsequent lane represents amplification for mRNA variants A–C.

Public databases (Ensembl, NCBI Genome Data Viewer) indicate the presence of at least 3 mouse AGRP mRNA transcripts. These transcript variants differ from each other at their 5′-UTRs only and share the same coding exons, suggesting different transcription start sites (TSSs). As these TSSs are separated by at least 10 kb (Figure 1A), it is likely that the presumed regulatory regions upstream of these TSSs will confer different, non-overlapping regulatory modes. We have denoted the proximal presumptive promoter as *Agrp-A*, containing binding sites for STAT3, FOXO1, and GR that confer sensitivity to fasting and leptin. The approximately 11 kb upstream presumptive promoter is termed *Agrp-B*, while the most distal presumptive promoter is termed *Agrp-C*. Interestingly, the public databases indicate that the 3 AGRP mRNA variants occur in nearly equal numbers, based on the numbers of intron-spanning reads from RNA-Seq data (Figure 1A).

We designed reverse transcriptase PCR (RT-PCR) assays to detect each AGRP mRNA variant using a variant-specific 5′ sense primer and a common antisense 3′ primer. Using adult mouse mRNA from hypothalamic blocks that included the median eminence, we readily detected amplicons for all 3 AGRP mRNA variants (Figure 1B), obviating the possibility that the variants are expressed only in early developmental stages.

### Regulation of expression of the AGRP mRNA variants by the bile acid taurocholate and leptin.

**Figure 2:**
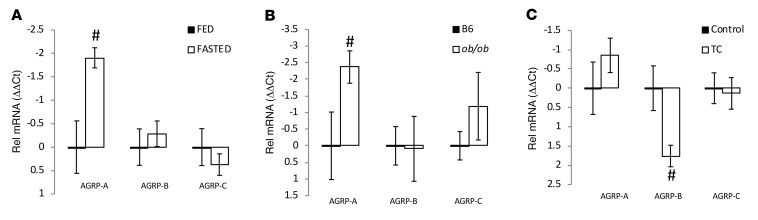
Regulation of AGRP mRNA variants by fasting, leptin, and the bile acid taurocholic acid. RNA from the hypothalamic blocks, including the median eminence and pars tuberalis, was used to detect and quantify the amounts of the 3 AGRP mRNA variants by quantitative RT-PCR with mRNA variant–specific assays. (**A**) Mice were fasted for more than 20 hours or fed ad libitum, and sacrificed between 10 am and 12 pm. (**B**) We compared the concentrations of AGRP mRNAs in leptin-deficient (*ob/ob*) male mice versus wild-type males. In **A** and **B**, note the greater amount of AGRP-A mRNA in fasted and *ob/ob* mice. (**C**) We used male mice that had been gavaged with taurocholate (TC) for 5 days with controls gavaged with saline. Note the reduced amount of AGRP-B mRNA in taurocholate-treated mice. All groups had 4–5 male mice except for **A** with 15 fed and 12 fed mice. ^#^*P* < 0.05, 1-tailed *t* test. Relative mRNA concentrations are reported as ΔΔCt values.

We went on to assess the changes in expression of these AGRP mRNA variants in response to physiological challenges by semiquantitative RT-PCR. We found that only AGRP-A mRNA was increased by fasting, while neither AGRP-B nor AGRP-C mRNAs were affected by fasting (Figure 2A). To further investigate this, we examined the amounts of the AGRP mRNA variants in leptin-deficient mice (*ob/ob*). Similarly to the fasting study, we found that only AGRP-A mRNA was increased in *ob/ob* hypothalamus (compared with wild-type hypothalamus) whereas neither AGRP-B nor AGRP-C mRNA was affected by leptin deficiency (Figure 2B).

We examined the potential changes of the AGRP mRNA variants’ hypothalamic expression (Figure 2C) after treatment with taurocholic acid, an endogenous bile acid FXR agonist that stimulates FGF15/19 expression in and secretion from enterocytes. After 5 days of taurocholate gavage, a time interval that we had previously determined affected AGRP mRNA concentrations (13), we examined hypothalamic content of the AGRP mRNA variants. In this case, taurocholate treatment reduced AGRP-B mRNA significantly (~80% reduction in comparison with control mice treated with saline), whereas neither AGRP-A nor AGRP-C mRNA abundance was altered.

### The presence of multiple Agrp promoters is conserved evolutionarily.

**Figure 3:**
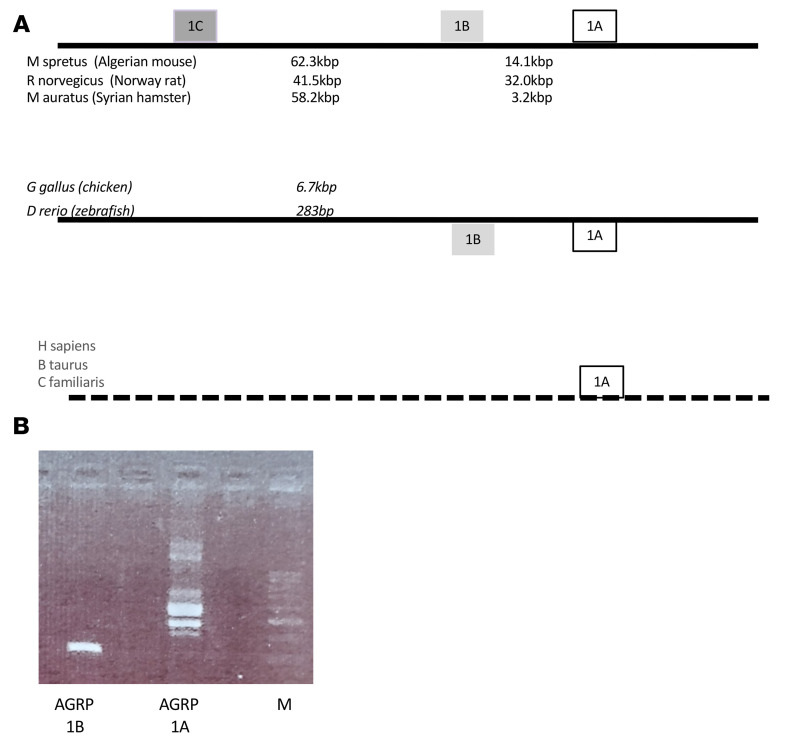
Evolutionary conservation of multiple AGRP mRNA variants. (**A**) Schematics of the *Agrp* gene structures and mRNA variants for multiple rodent species, model species of fish and bird, and large mammalian species (human, cattle, and dog). (**B**) Amplification of AGRP mRNA variants from chicken brain RNA. Note that while chicken AGRP 1B has 1 amplicon, chicken AGRP 1A has 2 major amplicons, the shorter one representing a splice variant that excises an intron fully contained within the first exon of the larger 1A transcript. The minor bands could represent other splice variants or heteroduplexes formed during the denature/anneal cycles during amplification.

The *Agrp* gene is evolutionarily conserved, being found in bony fishes, reptiles, amphibians, birds, and mammals. We explored the presence of multiple mRNA variants and multiple TSSs in model species (Figure 3) using the NCBI’s Genome Data Viewer (https://www.ncbi.nlm.nih.gov/genome/gdv/). Initially, we examined rodent species. The NCBI database indicated the presence of 3 transcript variants for *Mus spretus*, a mouse species that separated from *Mus musculus* about 1.5 million years ago (20). An expanded search in related rodent species showed 3 transcript variants and 3 TSSs in 2 rat species (*Rattus rattus* and *Rattus norvegicus*) and 2 hamster species (*Mesocricetus auratus* and *Cricetulus griseus*) with a similar spacing of exons and introns to the mouse. The guinea pig (*Cavia porcellus*) has 2 AGRP mRNA variants and 2 TSSs. Larger mammalian species (cattle, dog, and human) showed only 1 AGRP mRNA variant and 1 TSS, corresponding to the AGRP-A mRNA in rodents.

Given the widespread presence of multiple AGRP mRNA variants in mammals, we examined the public databases for AGRP mRNA variants in model species of fish and birds (Figure 3A). The zebrafish, *Danio rerio* (zebrafish), has multiple AGRP mRNA variants defining 2 TSSs. The chicken (*Gallus gallus domesticus*) expresses 3 AGRP mRNA variants that define 2 *Agrp* TSSs. We decided to experimentally determine the presence of AGRP mRNA variants in chicken brain. Using the same strategy of identifying 5′-UTR variants for AGRP mRNAs in mice, we designed primers corresponding to the 2 chicken AGRP mRNA 5′-UTRs with a common downstream primer. We were able to detect amplicons for both chicken AGRP mRNA variants (Figure 3B). In addition, the 5′-UTR primer located in exon 1A amplified 2 different-sized amplicons, which corresponded to the predicted sizes of alternatively spliced transcripts starting from the same TSS wherein an alternative intron is fully contained within an exon.

### An Agrp-B-FLP knockin/knockout mouse allele reveals expression in the pars tuberalis.

**Figure 4:**
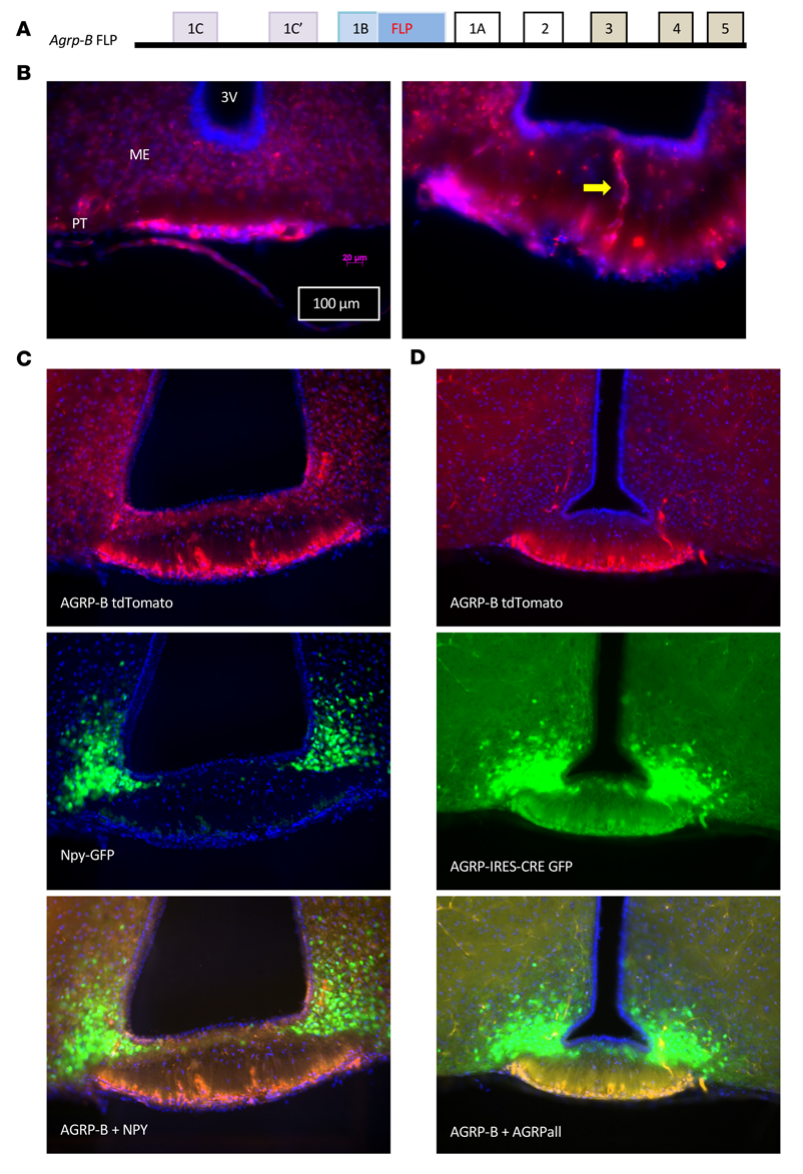
An *Agrp-B-FLP* knockin/knockout allele is expressed in the pars tuberalis. (**A**) Schematic representation of the *Agrp-B-FLP* knockin/knockout allele. The FLP recombinase coding sequence followed by a polyadenylation signal was introduced into exon 1B of *Agrp*. The polyadenylation signal acts as a transcription stop, preventing the production of AGRP-B mRNA that includes the *Agrp* coding exons. (**B**) Visualization of expression of *Agrp-B* with FLP-dependent expression of tdTomato (native fluorescence in red) from a female *Agrp-B-FLP FSF-tdTomato* mouse that is localized to the pars tuberalis (PT). A yellow arrow points to a cord of tdTomato cells that traverses the median eminence (ME) to end in the zone directly below the floor of the third ventricle (3V). Scale bars: 100 μm and 20 μm. (**C**) Imaging of a male *Agrp-B-FLP FSF-tdTomato Npy-GFP* mouse with GFP native fluorescence in green and tdTomato fluorescence in red. Note that the AGRP tdTomato signal is localized to the pars tuberalis while the Npy-GFP signal is localized to the arcuate nucleus (ARC). (**D**) Native fluorescence imaging from a male *Agrp-B-FLP Agrp-IRES-Cre Ai193* mouse with FLP activating tdTomato in red and Cre activating GFP in green. The AGRP-B tdTomato red signal is localized to the pars tuberalis, while the AGRP-GFP green signal is apparent in both the pars tuberalis and the ARC. The tdTomato and GFP signals in the pars tuberalis are co-incident. The GFP signal of the pars tuberalis is above the background signal but is much weaker than in the ARC. All sections were imaged at ×200 and mounted in DAPI-containing medium.

We generated an *Agrp-B-FLP* allele using CRISPR/Cas9 methodology (Figure 4A). The allele inserts the coding sequence of the FLP recombinase into the first exon/5′-UTR of *Agrp-B*. The allele is also effectively an *Agrp*-*B* knockout, since expression from the *Agrp-B* TSS drives FLP expression only due to the presence of a polyadenylation signal and a translational stop codon immediately after the FLP coding sequence, terminating transcription from the *Agrp-B* promoter. We verified this by quantitative RT-PCR of RNA isolated from the hypothalamic–median eminence blocks of *Agrp-B*-*FLP/Agrp-B-FLP* homozygotes. We were unable to amplify amplicons with the melting temperature observed in wild-type mice (85.6°C). Any amplifications were typically delayed by 9 cycles (ΔΔCt = 9.2 ± 2.2) with inconsistent melting temperatures within replicates and between samples, typical of aberrant amplification.

We used 2 FLP-dependent tdTomato reporter alleles (FSF-tdTomato and Ai193) to determine the site of expression of *Agrp-B* (Figure 4B). In *Agrp-B-FLP FSF-tdTomato* mice, we found that the tdTomato signal was in the pars tuberalis. The tdTomato-positive cells were small and ovoid, occurring as a band of cells ventral to the median eminence. In the anterior-posterior axis of the formalin-fixed mouse brain, the pars tuberalis, which extends 800 to 900 μm, appears as a flat sheet anteriorly that forms a tubular sheath around the pituitary stalk posteriorly. Occasionally, we would observe a centrally located branching cord of tdTomato-positive cells that traversed the inner and outer zones of the median eminence, approaching but not reaching the ependymal cells of the third ventricle. Examination of brain sections from *Agrp-B-FLP FSF-tdTomato Npy-GFP* mice (Figure 4C) revealed no expression of tdTomato in the hypothalamus, while the Npy-GFP signal was strongly expressed in the hypothalamic arcuate nucleus (ARC). However, there was strong expression of tdTomato in the pars tuberalis without any trace of Npy-GFP in the pars tuberalis. We also examined the degree of coexpression of *Agrp-B-FLP* and *Agrp-IRES-Cre* alleles using a dual recombinase reporter allele, Ai193, which can express EGFP after Cre recombination and tdTomato after FLP recombination (Figure 4D). In *Agrp-B-FLP Agrp-IRES-Cre Ai193* mice, we noted GFP and tdTomato fluorescence in the pars tuberalis that colocalized extensively. In contrast, the ARC exhibited intense GFP fluorescence with no evidence of tdTomato. As the pars tuberalis forms a part of the ventral portion of the third ventricle, typical dissections of the hypothalamus that incorporate medial structures such as the ARC, the ventromedial nucleus, and the dorsomedial nucleus would include the median eminence and the pars tuberalis, providing an explanation by which AGRP-B–expressing cells are included in the hypothalamic–median eminence block.

**Figure 5:**
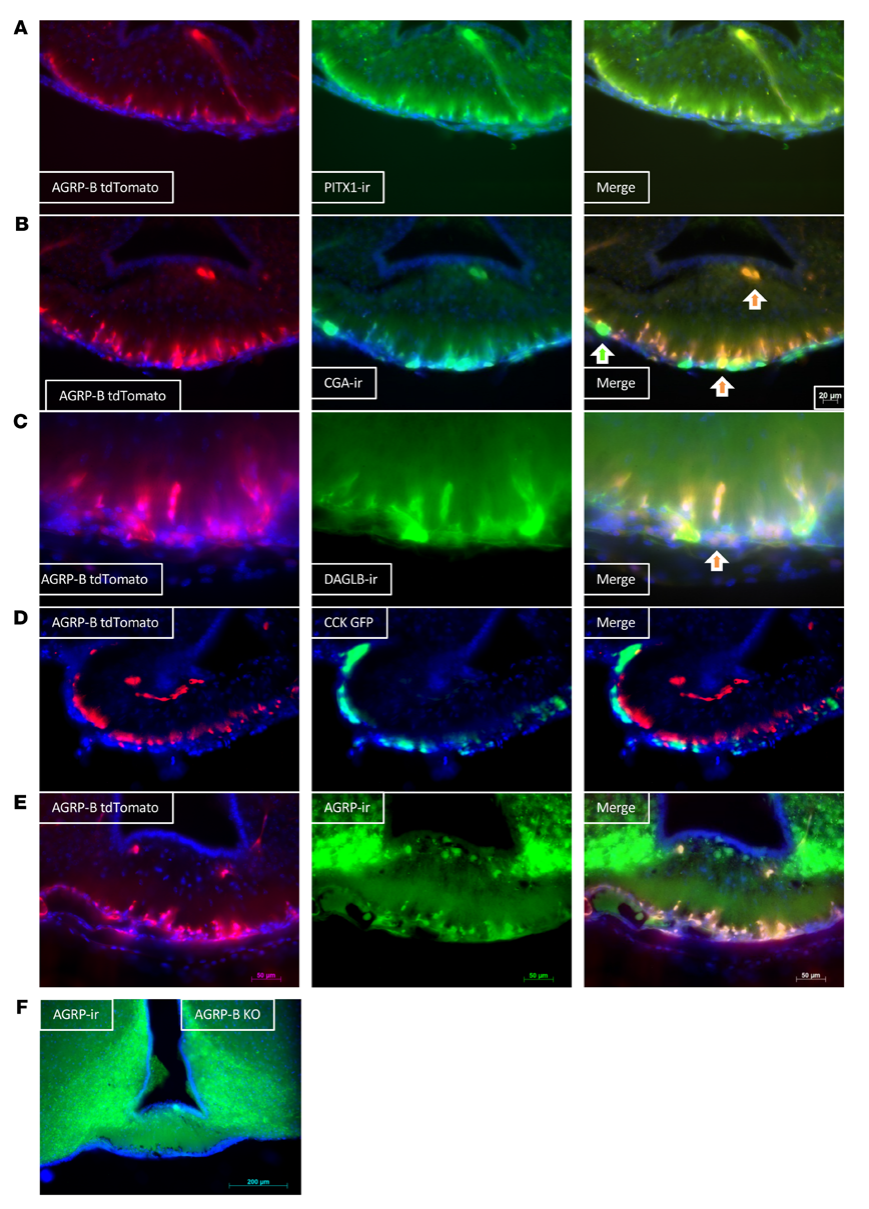
AGRP-B colocalizes with some markers for the pars tuberalis. (**A**–**C**) We performed immunostaining for PITX1, CGA, and DAGLB from sections of the brains of *Agrp-B-FLP Ai193* mice to determine colocalizations of these pars tuberalis markers with AGRP-B. There is perfect co-incidence between the PITX1 and AGRP-B tdTomato signals in **A**. In **B**, the AGRP-B tdTomato signal colocalizes with the CGA signal, although there are some CGA-positive cells that are not tdTomato labeled. Similarly, the DAGLB signal is co-incident with the AGRP-B signal, but there are some areas that are exclusively DAGLB-positive without AGRP-B. Orange arrows point to cells that show labeling for AGRP-B and CGA or DAGLB. (**D**) Native fluorescence from the brain of an *Agrp-B-FLP Cck-IRES-Cre Ai193* mouse with *Agrp-B*-*FLP* activating tdTomato (red) and *Cck-IRES-Cre* activating GFP (green). There is no colocalization of the tdTomato signal with the GFP signal. (**E** and **F**) Colocalization of AGRP-ir with the tdTomato fluorescence induced by Agrp-B-FLP with an *Agrp-B-FLP Ai193* male in **E** and an *Agrp-B-FLP/Agrp-B-FLP Ai193* (*Agrp-B*–KO *Ai193*) male in **F**. There is extensive colocalization of AGRP-ir and tdTomato fluorescence in both genotypes. All images were taken of 50 μm sections at ×200. Scale bars: 20 μm in **B**, 50 μm in **A**, **C**, **D**, and **E** and 200 μm in **F**.

The pars tuberalis is composed of endocrine cells and folliculostellate cells, with the endocrine cells expressing CGA, the common glycoprotein hormone α subunit. We found that the AGRP-B–expressing cells colocalized completely with PITX1-ir (ir = immunoreactivity) (18, 21) (Figure 5A), indicating a developmental origin from Rathke’s pouch that forms the pituitary. AGRP-B tdTomato fluorescence also colocalized with CGA-ir (Figure 5B). Occasionally, AGRP-B tdTomato fluorescence colocalized with CGA-ir in large acellular structures in the pars tuberalis (indicated by orange arrows in Figure 5B) and the median eminence, but there were cystic structures/follicles that were only CGA-ir (green arrow in Figure 5B). We also found that diacylglycerol lipase B (DAGLB), an enzyme involved in producing the endocannabinoid 2-arachidonylglycerol (2-AG), colocalized with AGRP-B tdTomato (Figure 5C).

Cholecystokinin (CCK) has been reported to be a pars tuberalis marker (19) that colocalizes with TSHB. We generated *Agrp-B-FLP Cck-IRES-Cre Ai193* mice to determine the possibility of coexpression of AGRP-B and CCK. However, AGRP-B did not colocalize with CCK (Figure 5D): the GFP-positive CCK-expressing cells formed a narrow layer of cells that were ventrally located to the inner layer of tdTomato-positive AGRP-B–expressing cells. The CCK-marked cells are consistent in size and location with the CGA-positive AGRP-B–negative cells observed in Figure 5B.

We confirmed that the AGRP-B–expressing cells also produced the AGRP neuropeptide (Figure 5E). An AGRP antiserum labeled cell bodies in several areas of the brains of *Agrp-B-FLP Ai193* mice: (a) pars tuberalis, (b) median eminence, and (c) ARC. The pars tuberalis cells coexpressed AGRP-ir and tdTomato, whereas some cells in the median eminence and all ARC neurons labeled only for AGRP-ir. We note that the images in Figure 4D and Figure 5E, comparing the distributions of AGRP-B cells and any cell expressing AGRP, are equivalent. Thus, the AGRP-B–expressing cells form a population of small neuroendocrine cells separate from the pars tuberalis CCK cells/thyrotropes.

In Figure 5F, we also show AGRP-ir in the pars tuberalis of an *Agrp*-*B*–KO (*Agrp-B-FLP/Agrp-B-FLPFLP Ai193*) mouse. There is no AGRP-ir signal in the cells that are labeled with tdTomato within the pars tuberalis of the *Agrp-B*–KO mouse, although there is a strong signal for AGRP-ir in the ARC.

### AGRP-B–expressing cells are found in the anterior lobe of the pituitary gland.

**Figure 6:**
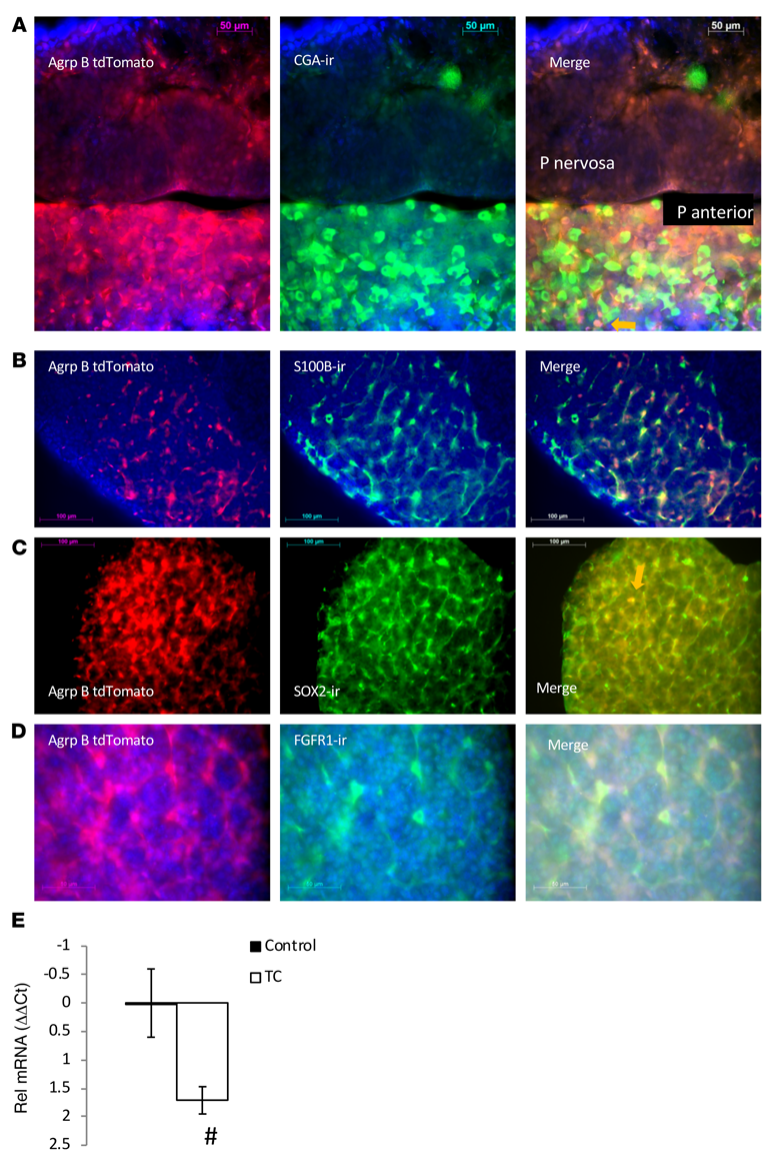
AGRP-B expression in the anterior lobe of the pituitary. (**A**) Cells expressing *Agrp-B* induced tdTomato fluorescence in the anterior pituitary of a female *Agrp-B-FLP Ai193* mouse (original magnification, ×400). There is extensive tdTomato fluorescence in star-shaped cells that are distinctly separate from discrete, rounded CGA-ir hormone-expressing cells. An orange arrow in the merged image points to a double-labeled cell (AGRP-B^+^CGA^+^). The pars nervosa has very weak, if any, tdTomato fluorescence. Scale bars: 50 μm. (**B** and **C**) Immunoreactivity for S100b (**B**) and SOX2 (**C**) in star-shaped cells of the anterior lobe that show extensive codistribution with the tdTomato label of a female *Agrp-B-FLP Ai193* mouse. Scale bars: 100 μm. (**D**) Extensive labeling of FGFR1-ir with tdTomato-labeled star-shaped cells in the anterior lobe of a male *Agrp-B-FLP Ai193* mouse. Scale bars: 50 μm. (**E**) A 5-day taurocholate (TC) treatment could significantly reduce AGRP-B mRNA in the pituitary of 2- to 3-month-old male mice (*P* < 0.02, *n* = 6 per group). ^#^*P* < 0.05, 1-tailed *t* test.

We examined the rest of the pituitary gland for *Agrp-B* expression (Figure 6), and we should note that the pituitary has not been previously evaluated for AGRP expression. The pituitaries of *Agrp-B-FLP Ai193* mice showed dense and extensive tdTomato signal found in star-shaped cells of the anterior lobe (Figure 6). The cells appeared to form a dense network, unlike the individually distinct distribution of CGA-ir–positive cells (Figure 6A), presumptive thyrotropes, and gonadotropes. Interestingly, unlike the AGRP-B–expressing cells in the pars tuberalis, which expressed CGA, the anterior lobe AGRP-B cells very rarely expressed CGA. We noted that these AGRP-B–positive and CGA-positive cells were rounded in shape, unlike the much more typical star-shaped AGRP-B–positive CGA-negative cells. The identification of AGRP-B cells as folliculostellate cells was confirmed with immunostaining for 2 markers of folliculostellate cells, S100B and SOX2. We found extensive colabeling of the AGRP-B tdTomato tag with both S100B (Figure 6B) and SOX2 (Figure 6C). Keeping in mind that SOX2 is a marker for progenitor cells (22), it is possible that the rare AGRP-B/CGA double-positive cells were derived from AGRP-B/SOX2 progenitors. We also found expression of FGFR1 on AGRP-B folliculostellate cells (Figure 6D), suggesting that FGF regulation of folliculostellate cells is a possibility. Based on this FGFR1 expression, we examined the regulation of AGRP-B expression in the anterior lobe by taurocholate. Measuring AGRP-B mRNA by quantitative RT-PCR, we found that AGRP-B mRNA was significantly reduced (Figure 6E) by a 5-day taurocholate treatment (ΔΔCt difference of 1.71, 3.3-fold reduction).

### The AGRP-B–expressing cells regulate glucose tolerance and insulin secretion.

**Figure 7:**
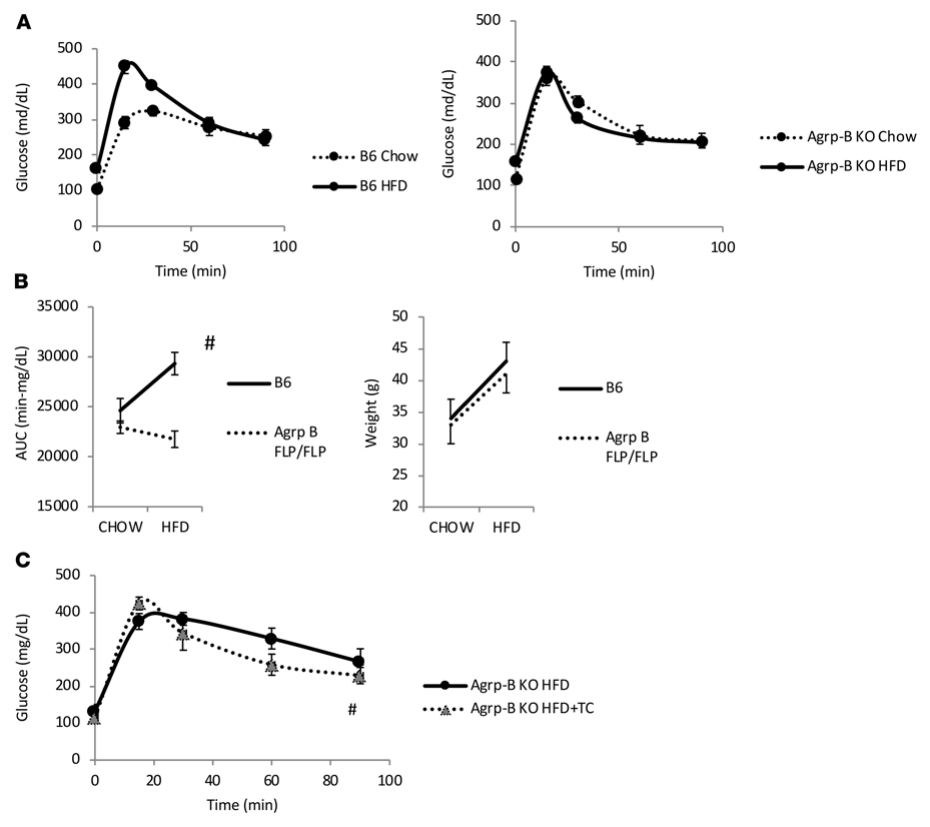
AGRP-B is involved in developing glucose intolerance during high-fat feeding. (**A**) Oral glucose tolerance tests (oGTTs) of *Agrp-B*–KO (*Agrp-B-FLP/Agrp-B-FLP*) male mice and control littermates on normal chow and a 60% fat by calorie diet (*n* = 5, 2–3 months old at the start of the study, 28 days on high-fat diet [HFD]). Mice were tested on chow at 2 months of age, placed on HFD for 4 weeks, and retested at 3 months of age. Note that mice of the 2 genotypes were not different when fed chow whereas only the control mice became glucose intolerant when fed the HFD. (**B**) Body weights at the start and conclusion of the study, with both groups of mice gaining equivalent body masses on HFD. Also shown are the areas under the curve (AUCs) for the 2 genotypes on chow and HFD, with only the control siblings increasing their AUCs during the GTT while the AGRP-B–deficient mice showed no alteration despite gaining weight. (**C**) The effect of taurocholate (TC) on *Agrp-B*–KO (*Agrp-B*-*FLP/FLP*) mice on HFD (*n* = 6 males, 50 days on HFD, 5 days of taurocholate treatment). There is a small but significant improvement in glucose tolerance after taurocholate gavage of *Agrp-B*–KO mice, based on an analysis of the AUCs. ^#^*P* < 0.05, 1-tailed paired *t* test.

As taurocholate suppressed the AGRP-B mRNA variant and improved glucose tolerance and GSIS, we sought to determine whether suppressing AGRP-B cells would cause improvements in glucose tolerance and GSIS. Initially, we examined the ability of animals lacking AGRP-B (*Agrp-B-FLP/Agrp-B-FLP*) to regulate glucose homeostasis. On normal chow, there was no difference between wild-type and *Agrp-B–*KO mice (data not shown). We then placed the mice on a high-fat diet known to induce glucose intolerance in C57BL/6J mice. The 2 groups of mice gained equivalent masses over the high-fat diet feeding period (4 weeks) (Figure 7B). However, it was clear that the AGRP-B–deficient mice were protected from glucose intolerance, based on their oral glucose tolerance tests (oGTTs) (Figure 7, A and B), as the areas under the curve (AUCs) for the AGRP-B–KO mice were not altered by high-fat feeding whereas the control C57BL/6J males on the high-fat diet were glucose intolerant. We also tested to see whether taurocholate treatment would be effective in *Agrp-B*–KO male mice after 7 weeks on a high-fat diet (Figure 7C), much longer than the duration for the mice in Figure 7A and Figure 7B. We found that taurocholate treatment did mildly improve glucose tolerance in mice without AGRP-B. This was not surprising since taurocholate acts on both FXR (found on enterocytes) and GPBAR1 on enteroendocrine cells (23). However, this study used *Agrp-B*–KO mice, in which AGRP-B was never expressed at any stage of development, with potential disruptions due to developmental effects. Thus, we sought to use a model wherein transient inhibition of AGRP-B–expressing cells occurred in adult mice to impute direct physiological relevance of the AGRP-B cells.

**Figure 8:**
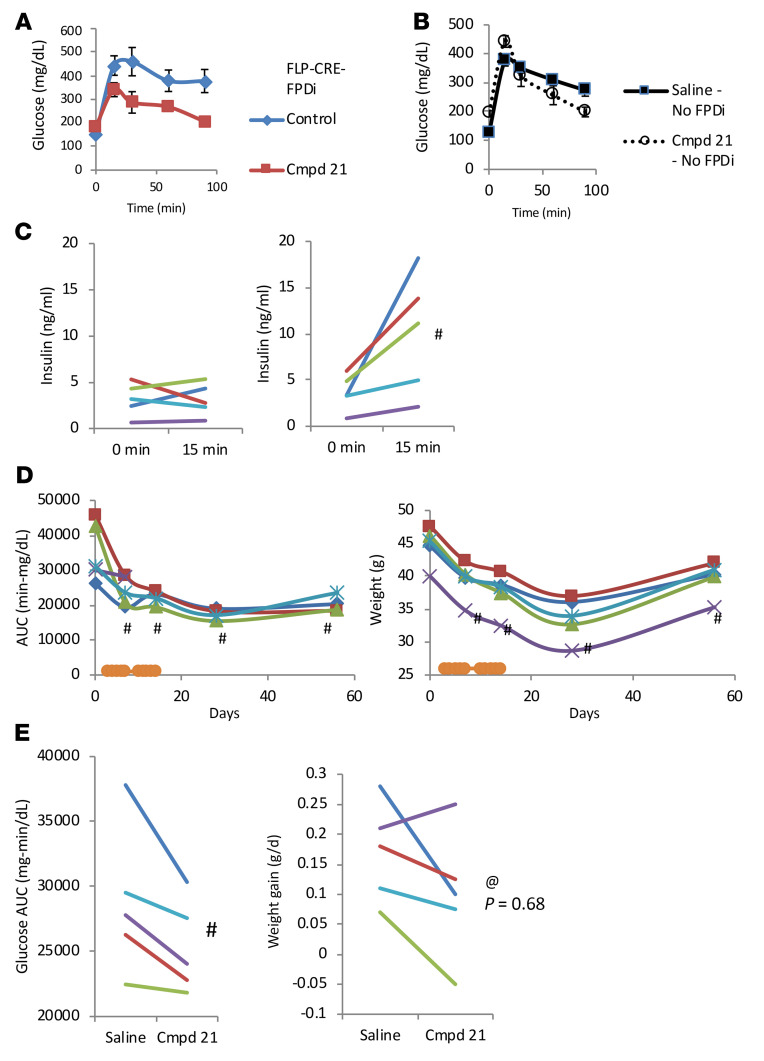
Acute inhibition of AGRP-B–expressing cells results in long-lasting improvement in glucose tolerance by restoring glucose-stimulated insulin secretion. (**A**) oGTTs after chemogenetic inhibition of AGRP-B cells with Compound 21 activation of hM4Di in *Agrp-B-FLP Agrp-IRES-Cre RC:FPDi* mice. Male mice (*n* = 5, 8–10 months old) on chow were tested before and after Compound 21 treatment for 5 days. Chemogenetic inhibition of AGRP-B cells caused reduction in glucose concentrations at all time points after glucose load. (**B**) FPDi-negative control littermate males were similarly tested before and during Compound 21 treatment (*n* = 3, 8–10 months old, on chow). Compound 21 had no effect on glucose tolerance. (**C**) Insulin concentrations during GTT at times 0 and 15 minutes after glucose load. Before Compound 21 treatment, mice had no apparent increase in insulin secretion, whereas Compound 21 treatment produced an excellent increase in insulin secretion upon glucose loading. (**D**) Results of an extended testing period for the same mice with an additional 5-day period of Compound 21 treatment followed by a washout of 6 weeks. In **B** and **C**, each mouse is represented by its own colored line and/or symbol. oGTTs and body weights were followed over the course of the extended testing. The additional Compound 21 treatment did not result in any additional improvement in glucose tolerance. At 2 weeks and 6 weeks after cessation of Compound 21, mice retained their improved glucose tolerance. Similarly, body weights remained below starting weights after Compound 21 treatment and remained at this reduced level after cessation of Compound 21. We also examined the impact of inhibiting AGRP-B cells in young male mice (on high-fat diet) (*n* = 5, 3 months old). (**E**) Younger male *Agrp-B-FLP Agrp-IRES-Cre RC:FPDi* mice also show improvement in oral glucose tolerance with chemogenetic inhibition of AGRP-B cells (4 weeks old at initiation of high-fat feeding). The males were on a 60% fat diet 4 weeks before glucose testing. Also shown are body weight gains during 4-day intervals before and during Compound 21 treatment. Four of the five animals showed a reduction in body weight gain (*P* = 0.68, 1-way paired *t* test). ^#^*P* < 0.05, 1-tailed paired *t* test. @ denotes the given *P* value of a 1-way paired *t* test.

We used intersectional genetics to express an inhibitory DREADD (hM4Di) in AGRP-B–expressing cells by introducing a dual recombinase-dependent hM4Di-expressing transgene (RC:FPDi) into *Agrp-B-FLP Agrp-IRES-Cre* mice. In these triple-transgenic mice, a DREADD agonist, such as Compound 21 or CNO, will activate the inhibitory hM4Di that is expressed in cells that express both *Agrp-B-FLP* and *Agrp-IRES-Cre*. We tested the triple-transgenic mice (*Agrp-B-FLP Agrp-IRES-Cre RC:FPDi*) (on normal chow) with and without Compound 21 for their oral glucose tolerance and insulin secretory response during the glucose load (Figure 8). After a 5-day treatment with Compound 21, a regimen that mimics the 5-day taurocholate gavage (13), we noted that the triple-transgenic male mice showed improved glucose tolerance (Figure 8A) and restored GSIS (Figure 8C), compared with the control state prior to Compound 21 treatment. These males were 8 months old and relatively obese at the time of testing, so their glucose tolerance was notably impaired before Compound 21 treatment. We treated the mice for a second 5-day period, but the mice did not exhibit further improvements in glucose tolerance. We continued monitoring glucose tolerance after cessation of Compound 21 treatment. Interestingly, improvement in glucose tolerance was maintained up to 6 weeks after termination of the Compound 21 treatment (Figure 8D). The data for each mouse are presented longitudinally in Figure 8, C and D. We also noted that the mice had reduced body weights after Compound 21 treatment that persisted up to 6 weeks after cessation of Compound 21 treatment (Figure 8E). This was a surprising result, although the DREADD-mediated inhibition of these cells would include the synthesis and secretion of any other secreted factors produced by AGRP-B cells, which would include endocannabinoids and any currently unidentified secreted factor. Control littermate males that did not express the hM4Di DREADD (*n* = 3; no *RC:FPDi*) showed no difference in glucose tolerance (Figure 8B) or body weight between control and Compound 21–treated states (median AUC was 27,607 min-mg/dL in the basal state whereas it was 27,457 min-mg/dL after 5 days of Compound 21 treatment). We also tested for circulating concentrations of corticosterone and found no difference between control mice and triple-transgenic mice after Compound 21 treatment (controls, 161 ± 57 pg/mL; triple-transgenic Compound 21–treated mice, 117 ± 45 pg/mL). This is an important point since AGRP is expressed within the adrenal cortex of rats (24) and AGRP expression is regulated by glucocorticoids (25). 

We tested a younger cohort of male mice that were placed on a high-fat diet at 4 weeks of age for 4 weeks with subsequent testing before and after chemogenetic inhibition of AGRP-B cells. The data show a statistically significant decrease in the AUC for glucose during an oGTT after Compound 21–mediated activation of hM4Di (Figure 8E). In addition, 4 of 5 mice showed a decrease in weight gain during Compound 21 treatment (Figure 8E). The body weight data are clearly different from those of the older cohort as the younger mice are still in their weight gain phase of growth (mean weight is 25.3 g) whereas the older mice, at 8 months old, are in the asymptotic phase of growth.

## Discussion

### Existence and detection of multiple AGRP mRNA variants.

To our knowledge, the existence of multiple AGRP mRNA variants has not been reported in the literature, although the genome databases report their presence based on RNA-Seq data and automated alignments. As we were unable to ascertain the developmental stage or the tissues of origin in which the variants were detected, we sought to obtain this information. Our RT-PCR assays readily detected all 3 of the AGRP mRNA variants in the adult mouse hypothalamic block. In retrospect, the tissue block inevitably contains the median eminence and the pars tuberalis, which form the ventral portion of the third ventricle, a defining feature for hypothalamic sections that contain the arcuate nucleus (ARC), the ventromedial nucleus, and the dorsomedial nucleus. One issue that arose during our studies was the lack of reports about AGRP in the pars tuberalis. However, careful evaluation of in situ hybridization images in one of the first published reports about AGRP (26) did reveal evidence of AGRP mRNA within the pars tuberalis and median eminence. Several items contribute to the lack of detection of AGRP outside the ARC: (a) AGRP immunoreactivity is difficult to detect in the cell body unless colchicine is used to prevent transport and release of the neuropeptide; (b) the GFP tag of the Npy-GFP transgene is almost always used as a convenient surrogate for AGRP cellular identity, while the Agrp-IRES-Cre transgene is used for opto- and chemogenetic manipulations; and (c) the pars tuberalis is a fragile structure and is frequently lost during standard tissue processing. For this study, we routinely embedded the hypothalamic block in a gelatin support matrix to preserve the anatomical integrity of the pars tuberalis/median eminence with the hypothalamus (see Methods). Furthermore, current single-cell RNA-Seq methods rely on sequence acquisition from the 3′ ends of transcripts, obviating the possibility of obtaining information from the 5′-UTRs of any RNA.

The 3 widely spaced TSSs for the mouse *Agrp* gene clearly suggest that the regulatory elements for the 3 promoter regions are unlikely to be shared. The proximal *Agrp-A* promoter/TSS has been reported to be regulated by STAT3 (27), FOXO1 (28), and GR (25, 29). We found that the AGRP-A mRNA variant is regulated by fasting and leptin, as would be expected for a promoter regulated by these energy balance–sensitive transcription factors. Neither AGRP-B mRNA nor AGRP-C mRNA was affected by fasting or leptin deficiency, indicating their independence from the regulatory influences of the *Agrp-A* region. We did find that AGRP-B mRNA is suppressed by treatment with taurocholate, a bile acid agonist of FXR and GPBAR (30). We have previously shown that the gut-to-brain axis involving taurocholate/FGF15 improved glucose tolerance in multiple models of obesity and diabetes (14), which was dependent on FGFR1 in AGRP-expressing cells (13). Our current data indicate that the AGRP-B–expressing cells of the pituitary are the responsible actors for regulating glucose homeostasis in the AGRP-mediated response to bile acid signaling.

The effect of inhibiting AGRP-B cells, which required 4 to 5 days of treatment to be manifested, appeared to be long-lasting. The improved glucose tolerance and reduced body weight of mice with chemogenetically inhibited AGRP-B cells are long-lasting, persisting for 6 weeks after cessation of the chemogenetic inhibition. It is possible that the effect is longer-lasting, which will require further testing. It has been hypothesized that the pars tuberalis acts as a switch between biological states (31, 32). We would note the lack of effect on body weight gain due to the *Agrp-B* KO and propose that inhibition of 2-AG secretion and/or some other secreted factors from AGRP-B cells might contribute to the reduction of body weight by the DREADD-mediated inhibition of AGRP-B cells.

### Evolutionary conservation of multiple AGRP mRNA variants.

We found multiple rodent species to have 3 *Agrp* TSSs and 3 AGRP mRNA variants. We also experimentally verified that the chicken has 2 *Agrp* TSSs and 3 AGRP mRNA variants. The functional significance of AGRP in birds is relatively unexplored and indeterminate, especially given the knowledge that avian leptin is not expressed in adipocytes but in the brain (reviewed in ref. 33). Nonetheless, we would note that the pars tuberalis thyrotrope was identified as a crucial player in reproductive photoperiodic time measurement using Japanese quail (34, 35). Similarly, the zebrafish is reported to have 2 *Agrp* TSSs and 2 AGRP mRNA variants. Unfortunately, many bony fish species have duplicated genomes (36) that make molecular analyses more complex than in diploid organisms. Given this conservation of multiple *Agrp* TSSs in bird and fish species, we were surprised to find that most mammalian species have only a single *Agrp* TSS and 1 AGRP mRNA, including *Homo sapiens*. However, there is strong sequence conservation of the upstream genomic regions between many mammals, including humans (37). The regions are approximately 20–30 kb upstream of the most proximal *Agrp* TSS, providing the possibility that long-distance regulation via chromatin looping (38, 39) could occur even in genomes of species that only contain 1 *Agrp* TSS.

In humans, AGRP is found in cerebrospinal fluid (CSF) and blood (40–42), so AGRP access to target cells is not limited by neuronal projections and is not limited to downstream neurons. The primate melanocortinergic neurons are situated in the infundibular nucleus, a horseshoe-shaped contiguous structure overlying the ventrally located median eminence (43, 44), rather than the 2 separate nuclei as found in rodents. The differences in proximity to the median eminence and cells contained therein (pars tuberalis thyrotropes and tanycytes) between the anatomical structures might obviate the necessity of a separate population of pars tuberalis AGRP cells in humans and primates.

### AGRP expression defines a previously mysterious pars tuberalis cell type.

Our data pointed to the clear distinction between AGRP-B–expressing cells and CCK-expressing cells in the pars tuberalis. The AGRP-B cells form a layer of small cells that abut the external zone of the median eminence, sometimes forming cords of cells that traverse the entirety of the median eminence and ending in the vicinity of the ventral portion of the third ventricle. The AGRP-B cells express CGA and PITX1, both of which are markers for neuroendocrine cells of pituitary origin. To further characterize the AGRP-B cells, we used a *Cck-IRES-Cre* allele. Single-cell RNA-Seq data have indicated coexpression of CCK with FSHB and TSHB (19). The CCK cells constituted an external layer of cells that formed a shell around the AGRP-B cells. Thus, these data indicate that the AGRP-B cells are a previously mysterious cell type of the pars tuberalis that is CGA positive but β subunit negative (45).

Interestingly, we found that the AGRP-B cells coexpress DAGLB, an enzyme that is necessary for the biosynthesis of the major bioactive endocannabinoid 2-AG. There are reports of endocannabinoid production within the pars tuberalis from endogenously expressed enzymes (46). Moreover, this endocannabinoid system appeared to respond to photoperiod and to be a likely contributor to seasonality (47). It is possible that endocannabinoids contribute to the physiological impact of AGRP-B pars tuberalis cells, potentially accounting for the different effects on body weight of the absence of AGRP-B and inhibition of AGRP-B–expressing cells.

The close proximity of these multiple cell types within the pars tuberalis could provide for the potential of cross-regulation as well as influences on the function of tanycytes (48, 49) in a manner similar to the pars tuberalis thyrotropes. Moreover, the presence of the adenohypophyseal portal system (50) permits the AGRP-B cells to influence target cells in both anterograde and retrograde directions, affecting pituicytes, hypothalamic neurons, and tanycytes.

### Expression of Agrp-B in the anterior pituitary.

We noted expression of *Agrp-B* in folliculostellate cells of the anterior lobe, showing extensive coexpression with S100B and SOX2 (51). We did find some rare cells showing coexpression of AGRP-B and CGA, suggesting that AGRP-B cells are potential progenitors of hormone-secreting cells of the anterior lobe. Folliculostellate cells are heterogeneous with potential roles as endocrine cell progenitors, support cells, and paracrine cells that secrete numerous factors that could synchronize/modulate secretion from endocrine cells (22, 52). Folliculostellate cells are also chemically and electrically connected via gap junctions, permitting rapid and long-distance communication across the span of the pituitary gland (53, 54). Lactotropes and prolactin gene expression (55) have been shown to be targets for melanocortins (56), mediated by MC3R (57) expressed in lactotropes. The quantity and nature of the AGRP released from folliculostellate cells remain to be determined, as folliculostellate cells do not have storage granules (51) and may not have the capacity for posttranslational processing of AGRP.

An unresolved issue remains regarding the contributions of the pars tuberalis and the anterior lobe AGRP-B cells to the improved glucose tolerance and body weight effects in our studies. Further work, potentially using various Cre lines (such as CGA-Cre and S100B-Cre) in combination with the *Agrp-B-FLP* allele to drive hM4Di in more restricted cell types, may define the cell type(s) responsible for the metabolic effects reported here. It is certainly possible both cell types contribute to the changes in glucose homeostasis and energy balance.

### A biological switch for different states of glucose homeostasis and energy balance.

Our most salient finding is the ability of the AGRP-B cells to respond to a diet-based signal from the gut whereas caloric stores/nutritional status do not appear to affect *Agrp-B* expression. The impact of AGRP-B cells requires several days to become evident, distinct from the rapid onset of hypothalamic AGRP/NPY neurons upon feeding, which occurs within seconds/minutes (1, 2). Furthermore, the long-term effects of inhibiting AGRP-B–expressing cells on body weight and glucose tolerance would suggest the presence of a metabolic switch that controls glucose homeostasis. At an organismal level, a consistent and plentiful supply of nutrients would entail adjustments to accommodate the influx of calories, of which increased insulin secretion is an appropriate response. Such a response is analogous to the impact of long day photoperiod length on reproduction that has been ascribed to the pars tuberalis thyrotropes (34, 35) which stimulates thyroid hormone production by tanycytes, which stimulate gonadotropin secretion and the reproductive axis. It is interesting that the effect is on insulin secretion rather than insulin sensitivity, as weight loss is typically associated with a reduction in insulin resistance. Mixed-nutrient meals cause the release of CCK, leading to the release of bile from the gallbladder (58). Bile acids traverse the intestine to interact with bile acid receptors, both FXR and GPBAR, to induce the secretion of FGF15/19 (59) and gut neuroendocrine hormones, such as GLP1 (60, 61). FGF15/19 acts on both the liver (to block bile acid synthesis) (62, 63) and the brain/pars tuberalis. Blocking antibodies against FGFR1 affect energy balance and substrate oxidation (64, 65), while their accumulation within the median eminence is a sign that the median eminence (and cells contained therein) is the likely mediator of the beneficial effects of blocking FGFR1 signaling. It is also possible that other FGFs are involved in this regulation, mimicking FGF15/19 (FGF21) (66, 67) or competing against it (classical FGFs). Endogenously produced classical FGFs could act in a paracrine manner, as they are bound to the heparan sulfated glycoproteins of the extracellular matrix at their sites of production (68). This is supported by our finding that short peptides based on FGF8 and FGF17 that prevent FGFR1 homodimerization and activation improve glucose homeostasis (13). The endocrine FGFs, FGF21, FGF23, and FGF15/19, circulate as they are not bound to the extracellular matrix (69). We have shown bidirectional effects on glucose homeostasis with genetic modification of FGFR1 expression in AGRP cells (13). Alterations of the composition of the extracellular matrix that affect local FGF binding/retention are likely to affect the binding and activity of the locally bound FGFs, a potential mechanism that could have long-term effects.

As the AGRP-B cells of the pars tuberalis and the anterior lobe are endocrine/paracrine in nature and not neurons, their targets for regulation could be melanocortin receptor–expressing cells that are accessible via the CSF or blood. Lactotropes and prolactin secretion are potential candidates for the glucose homeostatic effects modulated by AGRP-B cells, as prolactin can stimulate β cell proliferation (70, 71) and improve glucose-stimulated insulin secretion (70) by increasing glucokinase activity (72), as part of metabolic adaptations during pregnancy. As mentioned previously, lactotropes express MC3R and are regulated by melanocortins (55, 57). Indeed, the pars tuberalis has been reported to be a source of a prolactin secretagogue called tuberalin (73, 74). Furthermore, free dimeric CGA has been reported to increase lactotrope differentiation and prolactin release (reviewed in ref. 75). It has also been reported that pancreatic islets and insulin processing are directly regulated by melanocortins via MC4R (76).

The long-term reduction in body mass after induction of inhibitory hM4Di activity in AGRP-B cells suggests a profound shift in energy balance. Typically, acute weight loss is followed by hyperphagia and rapid weight regain. As hypothalamic AGRP/NPY neurons are central to a hyperphagic response in hypoleptinemic states, it is possible that AGRP/NPY neurons are targets of adenohypophyseal AGRP-B cells, potentially via MC3R (77), which is reported to be found in more than 95% of AGRP/NPY neurons. However, chemo- and optogenetic manipulations of hypothalamic AGRP/NPY neurons indicate that the modulation of food intake by these neurons is short-lived and is rapidly terminated upon cessation of the chemical or light stimulus (1, 2). Further investigations will be required to ascertain targets and mechanisms involved in the ability of AGRP-B cells to regulate body mass.

We have previously indicated the different time scales at which AGRP-A–expressing cells (minutes) and AGRP-B–expressing cells (days) manifest their metabolic effects. This difference in time scale would indicate that prior reports regarding the effects of hypothalamic AGRP/NPY neurons on feeding are unlikely to be affected by AGRP-B cells. However, studies involving chronic infusions of AGRP or chronic transgenic/virally mediated expression of AGRP may be confounded by effects on cellular targets of AGRP-B and AGRP-C. Knockout mouse studies on *Agrp* expression or ablation of AGRP cells could also be confounded by effects on non-hypothalamic neurons that express AGRP-B and AGRP-C. Determinations of AGRP mRNA might benefit from assays that distinguish between the various AGRP transcripts.

The observations that days-long treatments are required to activate the metabolic effects triggered by AGRP-B, which persist for weeks, point to sensing and effector mechanisms that operate over spans of days and weeks. These mechanisms may involve the proliferation of new cells, such as the proliferation of pancreatic β cells to increase insulin secretory function. In summary, we have (a) described 3 AGRP transcripts derived from multiple alternative promoters, (b) identified sites of expression for AGRP-B (one of these 3 transcripts), (c) determined the regulation of AGRP-B expression by bile acids, (d) generated a mouse allele to manipulate expression of AGRP-B, and (e) determined the ability of AGRP-B–expressing cells to modulate glucose homeostasis and energy balance over a span of several weeks.

## Methods

### Animals.

Mice were housed in a specific pathogen–free facility and provided with water and chow ad libitum. The facility maintained a constant temperature range between 24°C and 27°C with a light/dark regime of 14 hours on and 10 hours off with lights on at 6 am. Standard diet was PicoLab Rodent Diet 5053 (LabDiet). Some animals were fed a high-fat diet (D12492, 60% fat by calories, Research Diets). Sodium taurocholate (86339, Sigma-Aldrich) was given by gavage at 3 mg/kg (13). Compound 21 (5548, Tocris) was similarly gavaged at 1 mg/kg. Some transgenic mice had been maintained in-house for multiple generations from stock purchased at The Jackson Laboratory (Npy-GFP, JAX 006417; Agrp-IRES-Cre, JAX 012899) and had strain contributions from C57BL/6J, FVB/NJ, and 129. Other strains were recently obtained from The Jackson Laboratory: Ai193 (JAX 034111), FSF-tdTomato/Ai65F (JAX 032864), RC:FPDi (JAX 029040), Cck-IRES-Cre (JAX 012706). Mice carrying both *Agrp-B-FLP* and *Agrp-IRES-Cre* required parents carrying the opposing alleles to produce the compound heterozygotes. We did not encounter anomalous *Agrp-B-FLP* activity that resulted in germline deletion and transmission of deleted alleles, as was common with BAC transgenic *Agrp-Cre* mice (78). Genotyping was performed on ear clips with published protocols for each allele. Oral GTTs were performed as previously described (13).

### Generation of the Agrp-B-FLP–knockin allele.

The *Agrp-B-Flpe*–knockin founder mouse was generated by CRISPR technology (79) by the Albert Einstein College of Medicine Gene Targeting and Transgenic Facility. In brief, a guide RNA (gRNA) targeting exon 1 of *Agrp-B* (targeting sequence: TGGGACTGCAGACAACACAATGG) named Agrp gRNA 61-20 was designed with the online software Benchling (www.benchling.com) and generated by in vitro transcription. A homology-directed repair donor (HRD) plasmid, named AgrpFlpe HRD, was generated by SLiCE (80). AgrpFlpe HRD contains a DNA cassette consisting of Flpe-polyA signal flanked by homologous arms of around 2 kb at each side. Cas9 protein was purchased from PNA Bio. All the above CRISPR ingredients were mixed and injected into the fertilized eggs of C57BL/6 mice, and then the injected fertilized eggs were transferred to pseudopregnant CD1 female mice for producing pups. The resulting pups were genotyped to identify the positive founders (F_0_) carrying the expected modified alleles as follows: Agrp forward 1, TCCCCTGTCCTAGACCTTCC; Agrp reverse 1, AAAATGCCTTCCTCCCCTTA; Flpe reverse 1, TCAGTGATCTCCCAGATGCTT.

### Quantitative RT-PCR and hormone assays.

Total RNA was isolated by the guanidinium isothiocyanate extraction method. Total RNA (100–200 ng) was used to synthesize cDNA with RNase H– Mo-MuLV RT (New England Biolabs). Aliquots were used as templates for quantitation by SYBR Green fluorescence (Thermo Fisher Scientific.) during Taq DNA polymerase-mediated amplification with an Applied Biosystems Quant3. All primer pairs were synthesized by Integrated DNA Technologies and spanned at least 1 intron to prevent amplification of genomic DNA. Relative quantification was based on the ΔΔCt method using β_2_-microglobulin (B2M) as a loading control for each sample. Control means were set to 1.0 for comparison purposes. All amplification runs incorporated blank samples (no template) to assess false positive amplification. AGRP mRNA variants were amplified with the following primer sets: AGRP-A: mAGRP-A forward 3, CTGAAAGCTTTGTCCTCTGAAGCTG; mAGRP-ATG reverse, ACTCAGCAACATTGCAGTCAGCAT; AGRP-B: mAGRP-B forward 1, CCAGGCTATATAACAAAATCTGTGAG; mAGRP-ATG reverse, as above; AGRP-C: mAGRP-C forward 1, CTGCTGGAGAAGCAACGCAGGGAG; mAGRP-ATG reverse, as above; B2M: B2M forward, CAGTGGCTGCTACTCGGCGCTTC; B2M reverse, GCGTGAGTATACTTGAATTTGAGG.

Blood was collected from a nicked tail vein at the stated times. Serum was collected after centrifugation and used for hormone ELISA assays: insulin (Abnova) and corticosterone (Cayman).

Chicken total brain RNA (CR-201, Zyagen) was used for amplification of chicken AGRP cDNA amplicons as was done with mouse AGRP. Amplifications were performed with the following primer pairs: cAGRP 201: cAGRP 201 forward, ATAGGAAGCTGCTCCCATCCGCTC; cAGRP common reverse, CTGCGGTTCCAGCACGCTGCTTTTC; cAGRP 202: cAGRP 202 forward, CCCTGCAGAGCGGCGCCTGCAG; cAGRP common reverse, as above.

### Immunofluorescence.

Mice were euthanized with carbon dioxide inhalation and transcardially perfused with PBS supplemented with 5 mM EDTA as an anticoagulant followed by a fixative solution of PBS-formalin (equal volumes of PBS and 10% formalin in PBS). Both the brains and the pituitaries left in the skull were postfixed overnight with PBS-formalin and subsequently equilibrated in PBS. Hypothalamic blocks that included the median eminence were cut with a 5 mm brain matrix and embedded in 20% gelatin dissolved in PBS. Pituitaries were similarly embedded in gelatin before sectioning. The tissue blocks were sectioned with a Leica Vibratome in ice-cold PBS to obtain 50 μm sections. Free-floating sections were incubated/blocked in PBS plus 0.1% Triton X-100 (PBS-T) plus 5% donkey serum. Incubations with primary antibodies at 1:200 dilutions were performed in PBS-T plus 5% donkey serum in a cold room for 18–72 hours. We used the following primary antibodies: AGRP (guinea pig polyclonal, PA118414, Pierce Biotechnology), CGA (goat polyclonal, GtxHu-020-D, ImmunoReagents), DAGLB (rabbit polyclonal, NBP1-69662, Novus), FGFR1 (rabbit polyclonal, NBP2-24462, Novus), PITX1 (rabbit polyclonal, NBP1-88644, Novus), S100B (rabbit monoclonal, NBP2-53188, Novus), SOX2 (rabbit polyclonal, NBP2-24390SS, Novus). Secondary antibodies were goat anti–guinea pig–DyLight 488 (SA510094, Pierce Biotechnology), donkey anti-rabbit IgG–DyLight 488 (DkxRb-003-D488NHSX, ImmunoReagents), and donkey anti-goat–DyLight 488 (605-741-002, Rockland). Sections were washed in PBS-T three times and incubated with the appropriate secondary antibodies in PBS-T plus 5% donkey serum (1:200 dilutions). Sections were washed again three times with mild orbital shaking in PBS-T and mounted with Vectashield containing DAPI (H-1200-10, Vector Laboratories). Images were obtained with a Zeiss Axioplan microscope with the appropriate fluorescence filters. Native tdTomato and GFP fluorescence was imaged without the aid of immunostaining. Sections containing tdTomato were counterstained with DyLight 488–labeled secondary antibodies, whereas sections with GFP were counterstained with Alexa Fluor 568–, TRITC-, or Texas red–labeled secondary antibodies.

### Statistics.

The data are presented as mean ± SEM. Differences were significant with *P* values less than 0.05. One-tailed paired *t* tests were conducted where appropriate and are indicated in the legends.

### Study approval.

All animal studies were reviewed and approved by the Albert Einstein College of Medicine IACUC (protocol 00001186).

## Author contributions

SML, BI, FJ, AX, and JH performed experimentation and data acquisition and analysis and edited the manuscript. YY, GS, and YHJ performed experimentation, data interpretation, and imaging and edited the manuscript. SC conceptualized the study, performed imaging, analyzed data, and prepared the manuscript.

## Version 1 02/14/2023

In-Press Preview

## Version 2 04/03/2023

Electronic publication
